# Fluctuating selection strength and intense male competition underlie variation and exaggeration of a water strider's male weapon

**DOI:** 10.1098/rspb.2018.2400

**Published:** 2019-04-17

**Authors:** William Toubiana, Abderrahman Khila

**Affiliations:** Institut de Génomique Fonctionnelle de Lyon, Université de Lyon, Université Claude Bernard Lyon 1, CNRS UMR 5242, Ecole Normale Supérieure de Lyon, 46, allée d'Italie, 69364 Lyon Cedex 07, France

**Keywords:** weapons, scaling relationships, phenotypic variation, mating systems, reproductive fitness

## Abstract

Sexually selected traits can reach high degrees of phenotypic expression and variation under directional selection. A growing number of studies suggest that such selection can vary in space, time and form within and between populations. However, the impact of these fluctuations on sexual trait evolution is poorly understood. In the water strider *Microvelia longipes*, males display striking trait exaggeration and phenotypic variation manifested as extreme differences in the rear leg length. To study the origin and maintenance of this exaggerated trait, we conducted comparative behavioural, morphometric and reaction norm experiments in a selection of *Microvelia* species. We uncovered differences both in the mating behaviour and the degree of sexual dimorphism across these species. Interestingly, *M. longipes* evolved a specific mating behaviour where males compete for egg-laying sites, consisting of small floating objects, to intercept and copulate with gravid females. Through male–male competition assays, we demonstrated that male rear legs are used as weapons to dominate egg-laying sites and that intense competition is associated with the evolution of rear leg length exaggeration. Field observations revealed rapid fluctuation in *M. longipes* habitat stability and the abundance of egg-laying sites. Paternity tests using genetic markers demonstrated that small males could only fertilize about 5% of the eggs when egg-laying sites are limiting, whereas this proportion increased to about 20% when egg-laying sites become abundant. Furthermore, diet manipulation and artificial selection experiments also showed that the exaggerated leg length in *M. longipes* males is influenced by both genetic and nutritional factors. Collectively, our results highlight how fluctuation in the strength of directional sexual selection, through changes in the intensity of male competition, can drive the exaggeration and phenotypic variation in this weapon trait.

## 1. Introduction

Sexually selected traits represent some of the primary examples of intra- and inter-species phenotypic variation [1,2]. Males in both vertebrates and invertebrates are known to display extravagant phenotypes that differ in their nature, location, size and shape [1–4]. Such degrees of trait expression and variation among individuals are often associated with the type of mating strategy employed. In some systems, conflict over mating rate between the sexes can drive profound morphological modifications, such as male antennae in water striders that are used to grasp females during pre-mating struggles [5]. In other examples, such as in horned beetles or ruffs, males occur in discrete morphs associated with alternative mating strategies. Large males dominate territories, whereas small males tend to sneak [6–8].

An extreme form of variation lies in sexual characters displaying a continuum of trait expression with no distinguishable discrete morphs in the population [3,4]. A central prediction for these exaggerated traits to evolve is that only large individuals can afford to bear them as they are good indicators of body size, thus representing an honest signal for male quality [9–11]. Under this prediction, females will favour males with the highest trait expression, which imposes strong directional selection in favour of trait exaggeration [1]. In other situations, the exaggerated trait is used as a weapon in male–male competition, and its size is a good predictor for the outcome of the contest over access to females [12,13].

In these examples, sexual selection is thought to be directional and persistent over time [11,14]. These traits are also known to be subject to survivorship costs, which constrain their degree of expression resulting in a net stabilizing selection [4]. These observations raise important questions regarding the maintenance of phenotypic variation in natural populations [1,11,14–16]. A growing number of studies suggest that selection may fluctuate over time and space, and that environmental changes may influence the strength, direction and form of sexual selection [17–22]. These fluctuations in selection may, in turn, favour genetic variation and elevated plastic response observed in sexual traits, consequently influencing their variation and evolution [11,17,18]. Nonetheless, empirical studies have shown that the genetic and plastic influence on phenotypic variation could be highly variable from one species to another. For example, the three male discrete morphs of ruffs are mostly controlled genetically by different alleles in an inverted chromosomal region [23,24]. Major and minor morphs in dung beetles or wild turkeys, however, can mostly be recapitulated by changes in the environment such as nutritional intake or male competition [25,26]. Studies assessing the interplay between selection, genetics and plasticity, within the context of a changing environment, are therefore crucial to further the general understanding of the origin and maintenance of highly variable exaggerated sexual traits.

Here, we focus on a novel model system, the water strider *Microvelia longipes*, which displays a strong sexual dimorphism where males develop both longer and more variable rear legs than females [27]. The genus *Microvelia* (Heteroptera, Gerromorpha, Veliidae) comprises some 170 species of small water striders distributed worldwide and occupying various fresh water habitats including temporary rain puddles and stable large water bodies [27]. First, we reconstructed the phylogenetic relationships of five *Microvelia* species and compared their degrees of dimorphism, scaling relationships between leg and body length, and various aspects of mating behaviour. We report a clear association between the intensity of male competition and the evolution of trait exaggeration in *M. longipes* males. We then determined the fitness advantages of these exaggerated legs through fertilization success performed under selective conditions reflecting fluctuations in their natural environment. Finally, we assessed the contribution of the strength of sexual selection, genetic variation and phenotypic plasticity to the variation of exaggerated rear legs in *M. longipes* males.

## 2. Material and methods

### (a) Population sampling and culture

*Microvelia longipes*, *M. pulchella* and *M.* sp. populations were collected during fieldwork in French Guyana near Cayenne. *Microvelia americana* and *M. paludicola* were collected in North America. The bugs were maintained at 25°C and 50% humidity and fed on crickets.

### (b) Measurement of *Microvelia* species and statistics

Rear leg and body lengths of all *Microvelia* species were measured with a SteREO Discovery V12 (Zeiss) using the Zen software. All statistical analyses were performed in RStudio 0.99.486. Comparisons for the mean trait size and trait distributions were performed on raw data, whereas log-transformed data were used for scaling relationship comparisons. We used standardized major axis (SMA) regression to assess differences in scaling relationships (‘smatr’ package in R, [28]). Differences in intercepts were estimated using a Wald statistic test and we used likelihood ratio test for differences in slopes [28].

### (c) Behavioural observations and video acquisition

Male and female interactions of all *Microvelia* species were observed in a recreated small puddle, using local mud, and were filmed with a Nikon digital camera D7200 with an AF-S micro Nikkor 105 mm lens. Observations and video acquisitions were taken a couple of hours after the bugs were transferred to the puddle. In *M. longipes* and *M. pulchella*, male and female interactions were also observed in the field.

### (d) Microvelia phylogenetic reconstruction

The phylogenetic relationships between the five *Microvelia* species used in the behavioural assays were generated using the Geneious software v. 7.1.9 using plugins MrBayes v. 3.2.6 and PhyML v. 3.0, as described in [29]. The phylogenetic reconstruction was performed using 14 molecular markers. Electronic supplementary material, table S1 presents the identity and Genbank accession numbers for these markers. Phylogenetic reconstruction was performed using MrBayes v. 3.2.6 and PhyML v. 3.0 in Geneious 7.1.9 as described in [29].

### (e) Male competition assay

We generated five groups of three independent males from *M. longipes* laboratory population (*n* = 15 individuals). The males of each group were chosen for their differences in rear leg length (large, intermediate and small legs) and painted on their back. A male from each category fought five times with a male from the two other categories (total number of fights per male = 10).

### (f) Fight frequency assay

To compare the number of fights between males of *M. longipes* and *M. pulchella*, we isolated 25 adult males and females over a period of 2 days. Both sexes were then mixed together in the puddle during 30 min before observation. The number of fights on and outside floaters was counted during a period of 1 h (electronic supplementary material, table S2). We repeated the experiment the following day with the same males and females kept together overnight (electronic supplementary material, table S2). In order to correct for size differences between the two species, we calculated the number of fights in a reduced sample of 10 males and 10 females in *M. longipes* (electronic supplementary material, table S2). In all conditions, individuals were selected randomly (with respect to body and leg size) from the laboratory populations.

### (g) Artificial selection experiment

Individual males from the French Guyana natural population were selected for their absolute rear leg size and mated with a random female to initiate the successive sib–sib crosses. After 15 generations of sib–sib inbreeding, two populations selected for extreme phenotypes were amplified over two generations before phenotyping.

### (h) Condition-dependence experiment

First instar nymphs were collected just after hatching and individuals were reared in either poor or rich nutritional condition. In the poor condition, a hundred first instar nymphs of the long-legged inbred line were fed with 10 crickets per day during the first two nymphal instars, followed by only three cricket legs until adulthood. In the rich condition, 50 individuals of the same line were fed with 10 crickets per day over their entire nymphal development. In a second experiment, we tested the effect of condition in an independent set of individuals from the laboratory population. This experiment was performed on three replicates per condition, with 50 individuals per condition. Replicates were then pooled for the analysis. We started the poor condition by feeding the first two nymphal instars with eight crickets per day and then switched to one small cricket every 2 days until they reached adulthood. Individuals from the rich condition were fed during their entire nymphal development with eight crickets per day.

### (i) Microsatellite development

DNA from *M. longipes* was extracted from 10 males and females using the Genomic Genomic-tip 20/G DNA extraction kit from Qiagen. Using an ion-torrent sequencer machine, we generated 3.7 M reads with the median size of 317 bp. These sequences were used to identify reads containing microsatellite repeats using Exact Tandem Repeat Analyzer 1.0 software [30]. The primers for microsatellite amplification can be found in (electronic supplementary material, table S3).

### (j) Paternity test

To assess the fertilization success of long- and short-legged males, we collected six males from both the short- and the long-legged inbred lines, and put them together in an artificial puddle with 12 females from the long-legged inbred line. We conducted two treatments, each with four replicates, where we provided 20 floaters or three floaters in the puddle to create conditions with abundant and limiting egg-laying sites, respectively. On day 3, the parents were collected, their DNA extracted and the microsatellite of interest amplified using the protocol in electronic supplementary material, table S10 and sent for genotyping to Genoscreen, Lille, France. We then isolated the floaters and genotyped the nymphs that hatched from the floaters and those that hatched from the mud after adults and floaters were removed.

## 3. Results and discussion

### (a) Sexual dimorphism and scaling relationships in *Microvelia* species

**Figure 1.**
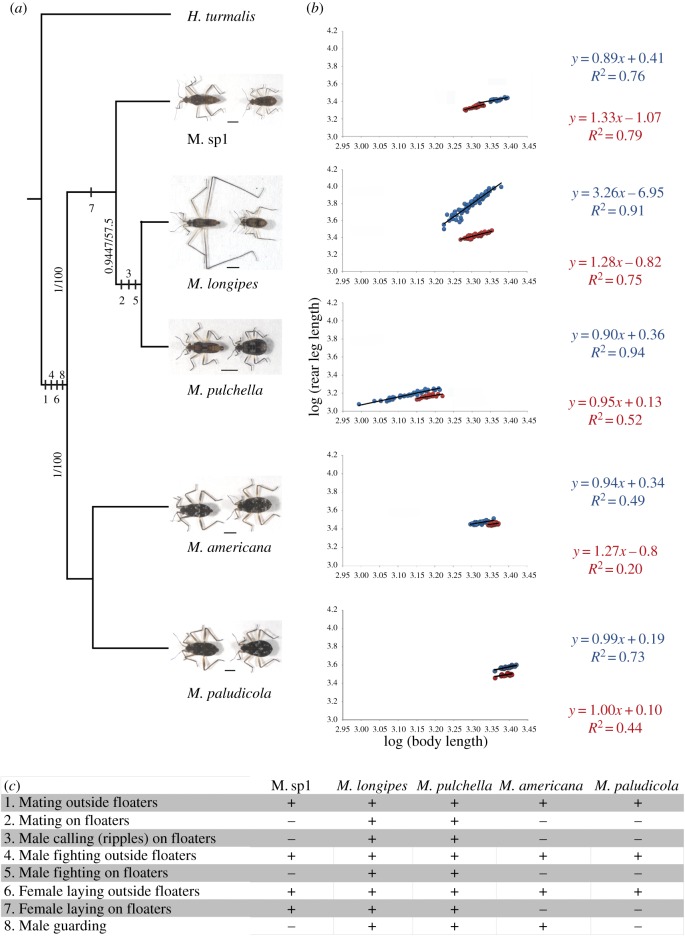
Diversity in leg sexual dimorphism and mating behaviours in *Microvelia*. (*a*) Phylogenetic relationships between five *Microvelia* species using maximum-likelihood and Bayesian analyses. Support values obtained after Bayesian posterior probabilities and 1000 bootstrap replicates, respectively, are shown for all branches. Pictures of males (left) and females (right) illustrate divergence in sexual dimorphism in the five *Microvelia* species. Scale bar represents 1 mm. (*b*) Scaling relationships of log-transformed data between rear legs and body lengths were estimated in males (blue) and females (red) of the five *Microvelia* species using standardized major axis (SMA) regressions. The equations and fitting (*R*^2^) of the regressions in males and females were indicated using the same colour codes. (*c*) Behavioural characters describing the mating system of the five *Microvelia* species. These characters were mapped onto the phylogeny based upon the parsimony criterion.

**Figure 2.**
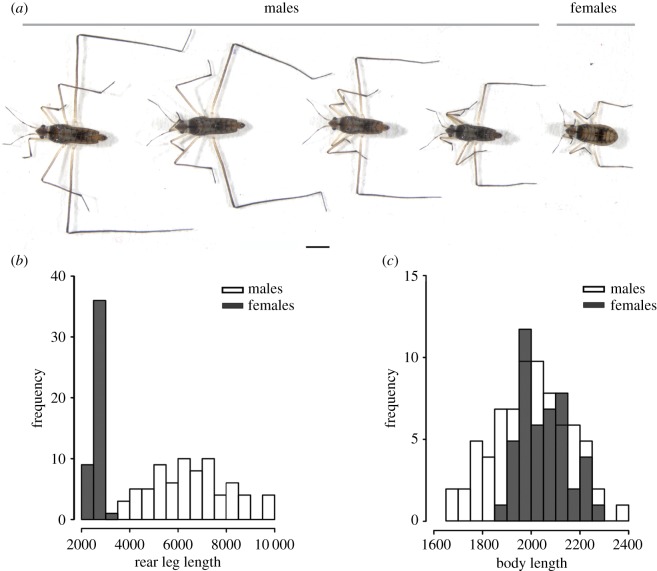
Phenotypic variation of rear leg exaggeration and body length in *M. longipes*. (*a*) Phenotypic variation of rear leg length in males and in a female. (*b*) Rear leg length and (*c*) body length distributions of males (white) and females (grey) from a natural population collected in French Guyana. Leg and body measurements are in micrometres.

We found a considerable inter-species variation in the degree of sexual dimorphism within the *Microvelia* genus (figure 1; electronic supplementary material, figure S1). Measurements of various body parts revealed dimorphism in average body length, leg length and the scaling relationship between these two traits (figure 1*b*; electronic supplementary material, table S4). In some species, such as *M. americana* and *M. paludicola*, the dimorphism in the leg and body length is small, whereas in others such as *M. longipes*, the dimorphism is most striking (figure 1*a*). The extreme leg elongation found in *M. longipes* males is associated with the evolution of hyperallometry where the allometric coefficient is significantly higher than 1 and reaches a value of 3.2—one of the highest known (figure 1*b*; electronic supplementary material, table S4) [31,32]. By contrast, *M. longipes* females and both sexes of all other species show isometric or near-isometric scaling relationships between leg and body length (figure 1*b*; electronic supplementary material, table S4). *Microvelia longipes* male legs are both significantly longer and more variable than female legs (figure 2*a,b*; see statistical tests in electronic supplementary material, table S4). By contrast, *M. longipes* body size is significantly more variable in males than in females, although females show slightly longer average body length (figure 2*a,c*; see statistical tests in electronic supplementary material, table S4). Despite these differences, leg and body lengths in both sexes assumed normal distribution (Shapiro tests: male third legs, *W* = 0.99; male bodies, *W* = 0.99; female third legs, *W* = 0.98; female bodies, *W* = 0.97; all *p* > 0.05; electronic supplementary material, table S5).

Finally, we found that the rear legs in males from three *Microvelia* species (*Microvelia* sp., *M. americana* and *M. paludicola*) bore prominent spikes that may function to grasp females during pre-mating struggles [33] (figure 1*a*; electronic supplementary material, figure S1). Overall, these analyses indicate that the evolution of hypervariable and exaggerated legs in *M. longipes* males is a derived trait resulting from the high variance in body length and the associated hyperallometric relationship with leg length. In *M. pulchella*, despite the high variation in male body length, the near-isometric relationship between leg and body length makes their legs less exaggerated and less variable than *M. longipes* males (figure 1*b*; electronic supplementary material, table S4). Moreover, the diversity of sexual dimorphism in leg morphology between *Microvelia* species does not seem to follow any phylogenetic signal (figure 1; electronic supplementary material, table S6), suggesting that variation in the ecology, behaviour or mating systems may play a role in the divergence of the sexes in these species.

### (b) Mating systems in *Microvelia* species

We characterized mating systems and sexual interactions in all five species to better understand the differences in sexual dimorphism (electronic supplementary material, figure S2). In nature, the *Microvelia* genus comprises species that occupy a wide variety of habitats [27]. Most species live near shore, in stagnant, large water bodies [27]. Some species, like *M. longipes*, *M. pulchella* or *Microvelia* sp., are gregarious and specialize in small temporary puddles filled with rainwater in tropical South America [27]. Behavioural observations both in the wild and in laboratory-recreated puddles revealed that *M. longipes* males are highly territorial and tend to aggressively guard floating objects such as small twigs or pieces of dead leaves (electronic supplementary material, figure S3). These are egg-laying sites where males signal to attract females, by vibrating their rear legs and pounding with their genitalia on the water surface to generate ripples (electronic supplementary material, videos S1 and S2). We hereafter refer to these objects as egg-laying floaters. When a female approaches the floater, the dominating male switches from signalling to a courtship behaviour. After inspecting the floater, she either leaves or mates without any resistance with the courting male, and immediately lays one to four eggs (*n* = 26 mating events) (electronic supplementary material, video S2). The male then initiates an aggressive guarding behaviour by turning around the egg-laying female and chasing other approaching males (electronic supplementary material, video S2). After the egg-laying female leaves and the male initiates another cycle of signalling on the same floater. During this entire process, other males constantly challenge the signalling male in an attempt to dominate the floater. During these contests, the dominant and the challenging male fight back to back by kicking each other with their rear legs until one of them is chased away (electronic supplementary material, video S2). We also observed that females could lay eggs in the mud at the margin of the puddle and that males attempt to mate outside floaters by jumping on females' back randomly in the puddle.

*Microvelia pulchella*, the sister species of *M. longipes* (figure 1*a*), is also found in small temporary puddles and displays a highly similar mating behaviour despite the lack of rear leg exaggeration (figure 1*c*). Males of *M. pulchella* compete for egg-laying floaters, fight with their rear legs and generate ripples to attract females. Like *M. longipes*, females of *M. pulchella* also lay their eggs on floaters and in the mud (electronic supplementary material, video S3 and figure S2). Despite the similarities in their mating behaviour, these two sister species display significant leg length differences, raising the question as to which factors drove the evolution of leg exaggeration in *M. longipes*.

In the three other species, *M. americana*, *M. paludicola* and *Microvelia* sp*.*, males possess grasping spines on their rear leg femurs (figure 1*a*; electronic supplementary material, figure S1) and actively harass females in an attempt to mate. Females consistently struggle through vigorous shaking, frequently resulting in the rejection of the male. Males of these three species also fight occasionally but the fights do not seem to result in the dominance of any particular localized resource (figure 1*c*; electronic supplementary material, video S4 and figure S2). *Microvelia americana* and *M. paludicola* females lay eggs exclusively at water margins, while *Microvelia* sp. females lay eggs either on floaters or at water margins, but not immediately after mating (figure 1*c*; electronic supplementary material, video S4 and figure S2). Altogether, these data show that the behaviour consisting of male contests using the rear legs is plesiomorphic among *Microvelia* species, predating the origin of the derived exaggerated legs in *M. longipes*. Male contests seem therefore necessary but not sufficient for the evolution of exaggerated weapons. Moreover, differences in egg-laying habits may have driven the diversity in male mating strategies and sexual dimorphism in the *Microvelia* genus. In small temporary habitats, eggs laid in the mud are at high risk of desiccation when water levels go down, and nymphs tend to drown at hatching when water levels go up, something we frequently observe in laboratory conditions (data not shown). Laying eggs on floating objects, which remain on the surface despite fluctuating water levels, is likely an adaptation to the fast-changing state of the habitat. Interestingly, male behaviour consisting of dominating these egg-laying floaters is observed only in species where females lay eggs just after mating, indicative of the high fitness value for the males who fertilize these eggs. This behaviour is also associated with a high body length variance in *M. longipes* and *M. pulchella* males (figure 1*b*), suggesting a link between body size variation and competition for oviposition sites.

### (c) Intensity of male competition in *Microvelia longipes* compared to *Microvelia pulchella*

**Figure 3.**
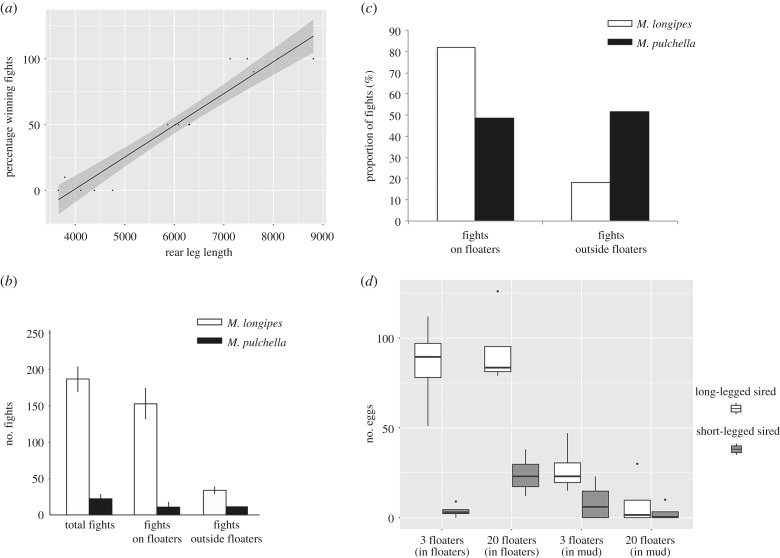
Selective pressures and reproductive fitness of leg exaggeration in *M. longipes* males. (*a*) Relationships between fighting outcome and male rear leg length. Winners correspond to males keeping the access to the egg-laying sites after the fights. Solid line represents the fitted regression from a generalized linear model (statistics in electronic supplementary material, table S7). (*b*) Number of fights during 1 h between *M. longipes* and *M. pulchella* (*n* = 50 individuals) on both floaters and outside floaters after 2 days of isolation. (*c*) Proportion of male fights both on and outside floaters for *M. longipes* and *M. pulchella* (*n* = 50 individuals) after 2 days isolation, (*d*) Fertilization success of large and small males and the contribution of egg-laying sites. Heterozygous eggs result from the siring of short-legged males (short-legged selected line) and females (long-legged selected line). Homozygous eggs result from the siring of long-legged males (long-legged selected line) and females (long-legged selected line).

In order to evaluate the contribution of exaggerated leg length to male mating success, we tested whether a correlation existed between male leg length and their ability to dominate egg-laying sites. We found increased rear leg length to be strongly correlated with the favourable fighting outcome, where the males with longer legs won 97% of the fights (*n* = 75 fights) and dominated the floater (GLM: *z*-value = 2.133, *p* < 0.05; figure 3*a*; electronic supplementary material, table S7). We also observed this male dominance over egg-laying sites in *M. pulchella,* which did not evolve leg exaggeration. We therefore hypothesized that the phenotypic differences in male legs between *M. longipes* and *M. pulchella* could be driven by differences in the intensity of male competition. When we measured male competition in standardized space conditions, we found that *M. longipes* males on average fought eight times more than *M. pulchella* males within 1 h (*t*-test: *t* = 15.18, d.f. = 4, *p* < 0.05; figure 3*b*; electronic supplementary material, table S2). Importantly, *M. longipes* males fought significantly more often on floaters than outside floaters, with 81% of the fights occurring on floaters (*t*-test: *t* = 9.37, d.f. = 4, *p* < 0.05; figure 3*c*; electronic supplementary material, table S2), whereas *M. pulchella* males fought randomly on or away from floaters (*t*-test: *t* = 0.15, d.f. = 4, *p* = 0.89; figure 3*c*; electronic supplementary material, table S2). Similar results were obtained when we repeated this experiment in standardized density conditions taking into account the size differences between the two species (electronic supplementary material, table S2). These data demonstrate that increased rear leg length in *M. longipes* males favours male dominance over egg-laying sites to better intercept gravid females. While both *M. longipes* and *M. pulchella* males intercept females and compete on those egg-laying sites, competition intensity for egg-laying sites is almost an order of magnitude higher in *M. longipes*. A primary difference between the ecology of these two species is that *M. longipes* specializes in rainwater-filled small puddles, while *M. pulchella* is a generalist that can be found in both temporary and more stable water bodies [34] (A.K. 2018, personal field observations). This difference in niche specialization has two major impacts on *M. longipes* population structure. First, *M. longipes* populations can reach very high densities confined in a small space, something we observed frequently in the wild and which is not the case for *M. pulchella*. Second, because the water level in the puddle can change rapidly (electronic supplementary material, figure S4), floaters represent the safest substrate in terms of survival of the progeny. This may explain why females bounce the floater up and down before they copulate and lay eggs (electronic supplementary material, video S2), and why *M. longipes* males are particularly aggressive in dominating these floaters. By contrast, *M. pulchella* occupy a more stable habitat, making floaters less critical and the survival of eggs in the mud more likely. The ecological conditions favouring high-density populations and floating objects as the more suitable egg-laying substrate may have at least contributed to the high competitiveness observed in *M. longipes*, and thus acted as a driving force for the evolution of the exaggerated leg length for use as a weapon. Both empirical and theoretical models suggest that population density can influence aggressiveness and the intensity of sexual selection [35], and our data show how increased competitiveness can drive secondary sexual traits to reach dramatic levels of expression.

### (d) Effect of exaggerated leg length on male reproductive fitness in *Microvelia longipes*

Post-mating competition is widespread in insects [36], including water striders [37,38], and can strongly alter the outcome of pre-mating strategies [36,39]. Field observations also indicate that the state of the habitat occupied by *M. longipes* can fluctuate rapidly and, sometimes, the water can evaporate entirely in days (electronic supplementary material, figure S4). Moreover, the available amount of resources that can serve as egg-laying substrates is highly variable from one puddle to another and therefore can also fluctuate with water level (A.K. 2018, personal observations from the field). We hypothesized that these rapidly changing conditions will influence competition and mating success across the distribution of male phenotypes. To test this hypothesis, we conducted paternity tests using *M. longipes* homozygous lines for distinct microsatellite markers that can reveal the identity of the parents (see Material and methods for more details). We set the experiment such that heterozygous progeny could only originate from eggs fertilized by small males. Because egg-laying floaters represent the primary resource that males dominate to intercept gravid females, we designed a first treatment where floaters were limiting (three floaters for six large and six small males) and another treatment where floaters were abundant (20 floaters for six large and six small males). We also genotyped the progeny from eggs laid in the mud to determine the mating success of different male phenotypes in contexts other than the dominance of floaters. In all replicates of each treatment, females laid significantly more eggs on floaters regardless of whether floaters were abundant (91% of a total of 512 eggs) or limiting (71% of a total of 500 eggs) (abundant floaters: *t* = 5.63, d.f. = 6, *p* < 0.05; limiting floaters: *t* = 3.02, d.f. = 6, *p* < 0.05; figure 3*d*; electronic supplementary material, table S8). However, they also laid on average three times more eggs in the mud when floaters were limiting, although this difference was not statistically significant (*t* = 1.56, d.f. = 6, *p* = 0.17; electronic supplementary material, table S8). In the condition where floaters were limiting, small males fertilized 4.6% (15 eggs of a total of 357 eggs) of the eggs laid on floaters and 25% of the eggs laid in the mud (35 eggs of a total of 143 eggs) on average (GLM: *z*-value = 5.903, *p* < 0.05; figure 3*d*; electronic supplementary material, table S8). This suggests that when the dominance of floaters by small males is limited, they primarily achieve egg fertilization by mating outside floaters. In the condition of abundant floaters, the proportion of eggs fertilized by small males on floaters increased significantly to 19% (96 eggs of a total of 468 eggs) (figure 3*d*; electronic supplementary material, table S8), while that outside floaters remained unchanged (11 eggs of a total of 44 eggs) (figure 3*d*; electronic supplementary material, table S8). In contrast to the treatment with limiting floaters, here the number of eggs fertilized by small males is almost nine times higher on floaters than in the mud (GLM: *z*-value = −3.547, *p* < 0.05; figure 3*d*; electronic supplementary material, table S8). These results show that small males can sire significantly more progeny when egg-laying sites are abundant but can also mate outside these egg-laying sites when floaters are limiting. Although we cannot exclude the possible effect of assortative mating, these results indicate that sexual selection is strong in favour of large males with long legs but can become relaxed in conditions where egg-laying sites are abundant. Rapid changes in water level and high heterogeneity between puddles are intrinsic to the life history of this species and are expected to cause variation in the amount of accessible egg-laying floaters over time and space. This fluctuating selection is therefore likely to influence the strength of competition and mating success and contribute to the high phenotypic variation found in *M. longipes* natural populations.

### (e) Environmental and genetic contributions to male rear leg variation

**Figure 4.**
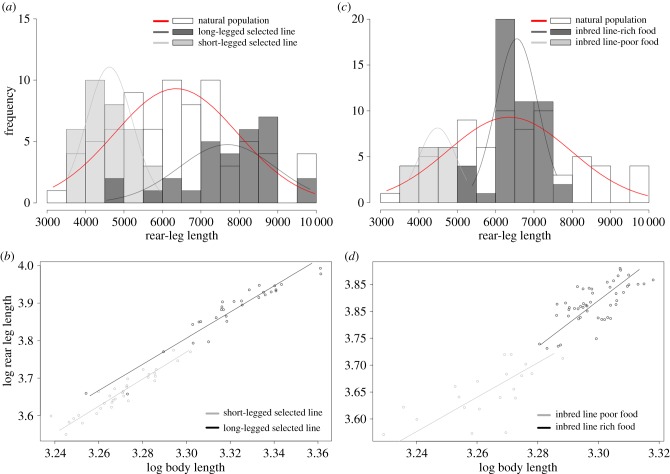
Environmental and genetic contributions to rear leg length variation in males *M. longipes*. (*a*) Rear leg length distributions of adult males from natural population (white) and from two inbred lines that were selected for short (light grey) or long (dark grey) rear legs under rich condition. Normal curves were fitted to each distribution after testing for normality (electronic supplementary material, table S5). (*b*) Scaling relationships of log-transformed data between rear legs and body lengths estimated in males from two inbred lines selected for short (light grey) or long (dark) rear legs under rich condition, using SMA regressions. (*c*) Rear leg length distributions of adult males from natural population (white) and from an inbred line that developed under poor (light grey) and rich (dark grey) conditions. Normal curves were fitted to each distribution after testing for normality (electronic supplementary material, table S5). (*d*) Scaling relationships of log-transformed data between rear legs and body lengths estimated in males from an inbred line that developed under poor (light grey) and rich (dark) conditions, using SMA regressions.

To test the relative contributions of genetic variation and phenotypic plasticity to male phenotypic variation, we artificially selected large and small male lines, generated through 15 successive sib–sib crosses from a natural population. The large and small male lines showed a shifted distribution of male leg length towards the respective extreme phenotypes of the distribution (figure 4*a,b*). In these two lines, there is a significant difference in absolute and relative leg length (*t*-test: *t* = 22.21, d.f. = 85.266, *p* < 0.05; Wald statistic test: *W* = 16.52, d.f. = 1, *p* < 0.05; electronic supplementary material, table S9), but the allometric coefficient remained unchanged (likelihood ratio test: likelihood ratio statistic = 0.17, d.f. = 1, *p* = 0.68; figure 4*b*; electronic supplementary material, table S9). This shows that genotypic variation contributes to the variation in both rear leg length and body size.

Next, we tested the reaction norm of one of these inbred lines and a laboratory population in poor and rich nutritional conditions. Despite the near identical genotype, individuals from the inbred line reared in poor condition developed shorter legs than individuals reared in rich condition such that the distributions of the two treatments were almost non-overlapping (*t*-test rear leg length: *t* = 15.374, d.f. = 39.232, *p* < 0.05; figure 4*c,d*; electronic supplementary material, table S9). Importantly, this difference in leg length between the two treatments resulted mostly from differences in overall body size (*t*-test body length: *t* = 10.5643, d.f. = 25.274, *p* < 0.05) but not in the scaling relationship as we failed to detect any significant difference in the allometric coefficient or the intercept between rich and poor conditions (likelihood ratio test: likelihood ratio statistic = 1.932, d.f. = 1, *p* = 0.16; Wald statistic test: *W* = 1.69, d.f. = 1, *p* = 0.19; figure 4*d*; electronic supplementary material, table S9). A similar result was obtained when we tested condition dependence in a laboratory population where no specific selection has been applied, although a small but significant difference in intercept was detected between the two conditions (Wald statistic test: *W* = 7.214, d.f. = 1, *p* < 0.05; electronic supplementary material, figure S5 and table S9). This difference was nonetheless not significant when using a linear model (ANCOVA, *F*_1,88_ = 2.6202, *p* = 0.1076). We therefore conclude that, in *M. longipes*, body length is highly dependent on nutritional condition. However, the scaling relationship between leg length and body length shows little, if any, condition dependence. Altogether, these results suggest that male leg length variation in nature results from the contribution of both genetic variation and strong condition dependence. The fluctuations in the number of egg-laying floaters, combined with phenotypic plasticity, are expected to result in the maintenance of a certain degree of genetic variation in the population through the incomplete removal of alleles of small leg and body size. However, episodes of relaxed selection are not only known to increase genetic variation in the population, but also to favour the evolution of reaction norms and therefore increase phenotypic plasticity [40,41].

## 4. Conclusion

This study illustrates how various ecological factors influence the intensity of sexual selection and ultimately the mechanisms and patterns of phenotypic variation. In the genus *Microvelia*, mating systems are diverse and are likely to influence the diversification of male-specific secondary sexual traits used in pre-mating copulatory strategies. The intense male competition to dominate egg-laying sites in *M. longipes*, unlike other *Microvelia* species, underlies the evolution of exaggerated leg length used as a weapon. Dominating males that intercept and copulate with gravid females on egg-laying sites gain a significant increase in their reproductive fitness by siring the majority of the eggs. This intense selection on increased leg length can, however, be relaxed when egg-laying sites are abundant, thus allowing small males to fertilize a significant number of eggs. We have also shown that plasticity in response to nutritional condition along with genetic variation both contribute to the high phenotypic variation we observed in body and leg length. It is possible that fluctuating selection, combined with phenotypic plasticity, facilitates the dramatic increase and maintenance of phenotypic variation in *M. longipes* compared to other *Microvelia* species. It is also important to note that the fluctuating selection described here (availability of egg-laying floaters) is independent of the individual condition. Therefore, its influence on phenotypic variation cannot be the consequence of a pre-existing increase in condition dependence, as it would be the case for fluctuating selection based on food resources, for example. Altogether, these results point to two ways in which alleles for small male body and leg size will be maintained in the population. First, because small males can sire a significant number of progeny due to possible episodes of relaxed selection. Second, because males with allelic combinations for low trait expression can develop larger body and leg size if they experience higher nutritional condition during development. Therefore, condition dependence causes a nonlinear relationship between genotypes and phenotypes, making directional selection less efficient in depleting genetic variation. This fits what Cornwallis & Uller [18] refer to as a ‘feedback loop between heterogeneity, selection and phenotypic plasticity’.

The findings outlined here open important research avenues to gain a general understanding of how sexual selection can impact phenotypic evolution. *Microvelia longipes* as a new hemimetabolous insect model with an exaggerated secondary sexual trait offers the opportunity to complete the substantial literature in holometabolous insects such as beetles or various flies [4,10,42,43]. Males of many species of water striders employ water surface ripples as mating calls, and it is unknown whether females can deduce the size of the male from the ripple pattern and whether this would influence female choice [44,45]. The ease of rearing and the relative short generation time make *M. longipes* a powerful future model to study the extent to which genetic variation and environmental stimuli influence gene expression and ultimately phenotypic variation.

## Supplementary Material

Figures 1-6

## Supplementary Material

Table 1-9

## Supplementary Material

Video S1

## Supplementary Material

Video S2

## Supplementary Material

Video S3

## Supplementary Material

Video S4

## Data accessibility

Data available from the Dryad Digital Repository: https://doi.org/10.5061/dryad.d4s8357 [46].
