# Supplementary material for: Fluctuating selection strength and intense male competition underlie variation and exaggeration of a water strider's male weapon
Source: Proc Biol Sci. 2019 Apr 17;286(1901):20182400. doi: 10.1098/rspb.2018.2400 (PMC6501938; doi:10.1098/rspb.2018.2400)
Supplement: Figures 1-6 [file rspb20182400supp1.pdf]

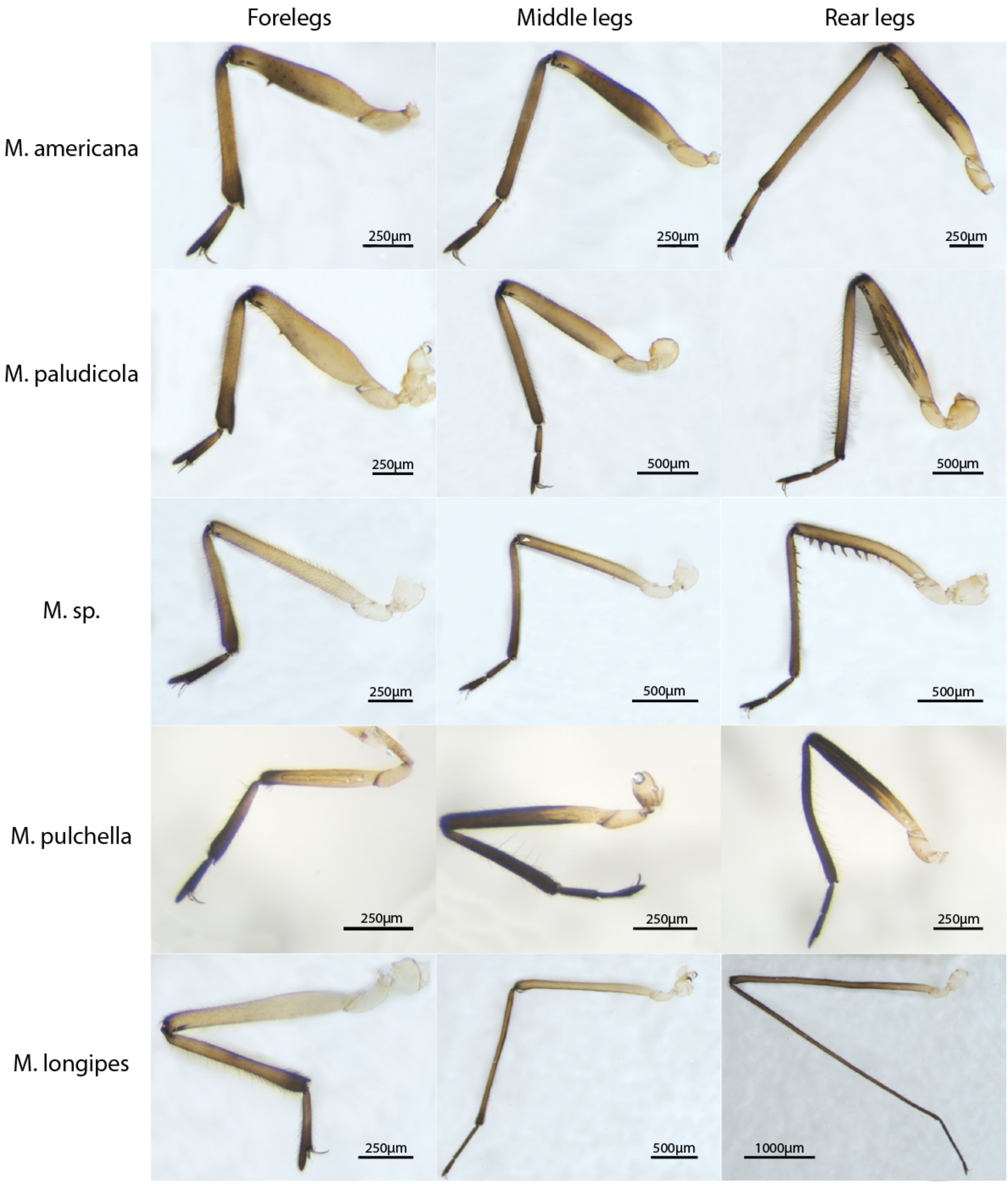

Supplementary figure 1: Comparative morphology of the legs across *Microvelia* species sample in figure 1. Note the presence of grasping traits on male legs in *Microvelia americana*, *Microvelia paludicola* and *Microvelia sp*. These traits are absent in *Microvelia longipes* and *Microvelia pulchella*.

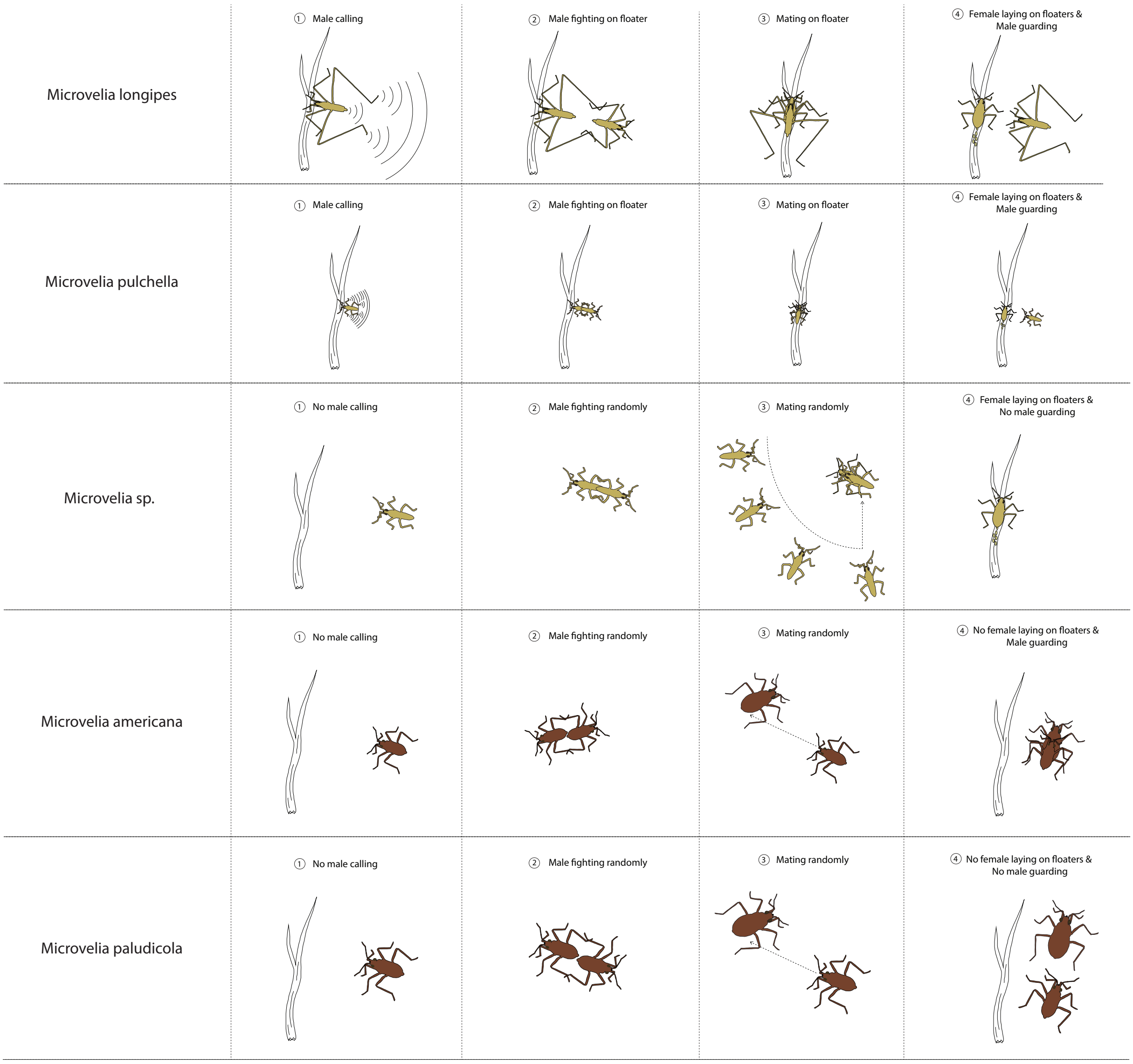

Supplementary figure 2: Schematic summary representation of the mating systems in the five *Microvelia* species

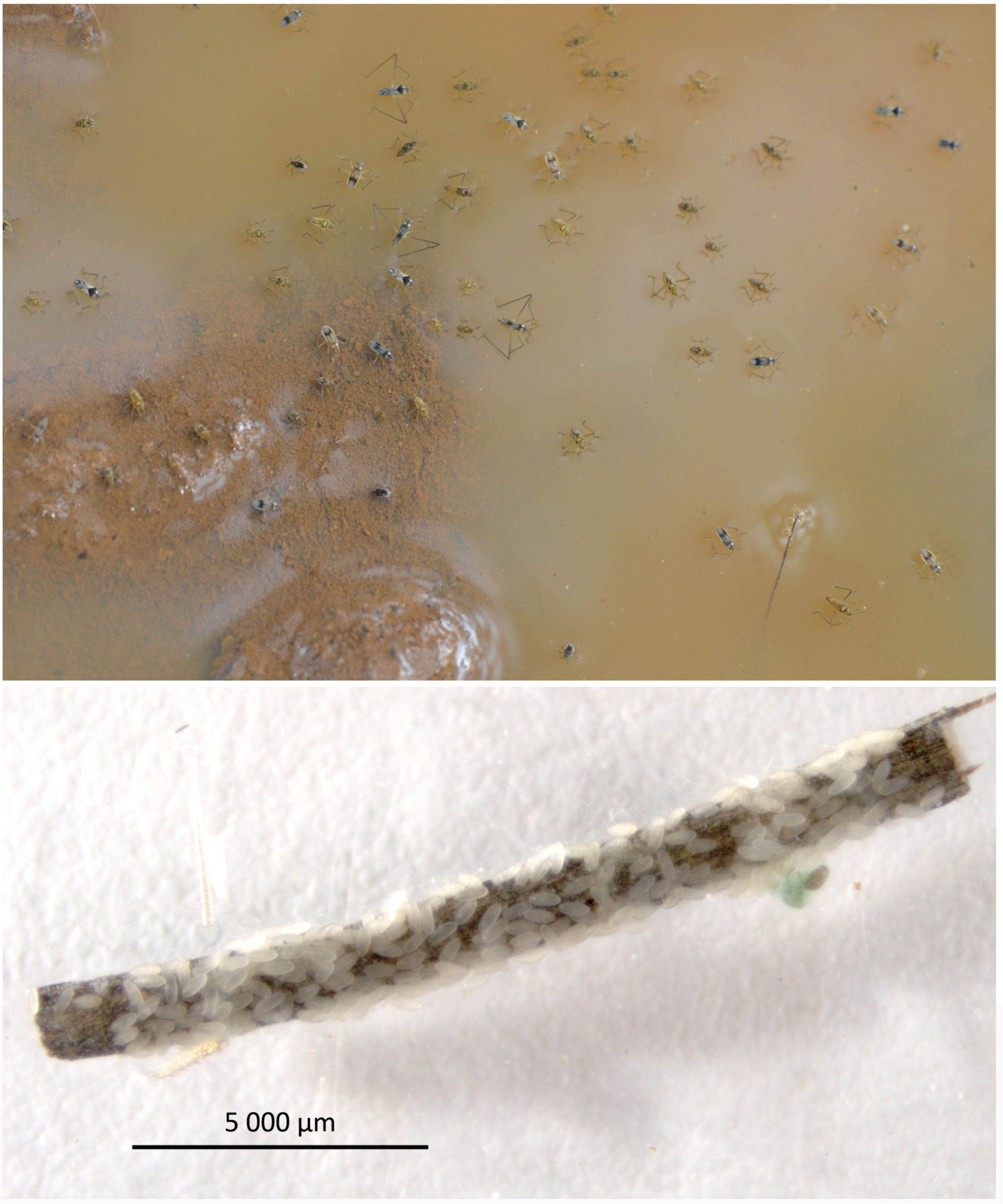

Supplementary figure 3: *M. longipes* natural habitat. Top panel: Example of rain-filled puddle in French Guyana in Crique Patate near Cayenne where *M. longipes* population was collected. Middle panel: Zoom on the floating substrates deposited on the water surface of the puddle. Bottom panel: Example of floater full of *M. longipes* eggs. Scale bar represents 5mm.

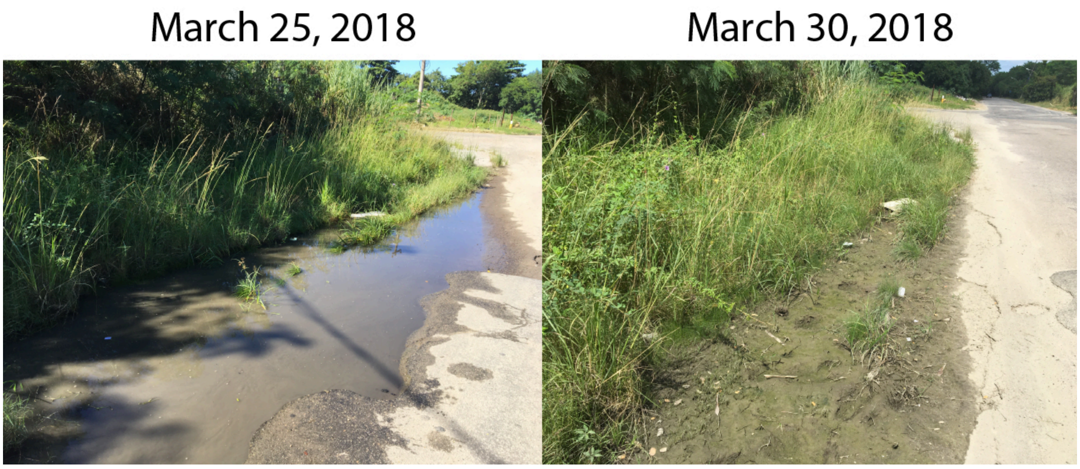

Supplementary figure 4: Fluctuating *Microvelia longipes* environment. Pictures of a rain-filled puddle in Rio de Janeiro where a *M. longipes* natural population was collected. The puddle dried out entirely in a period of five days.

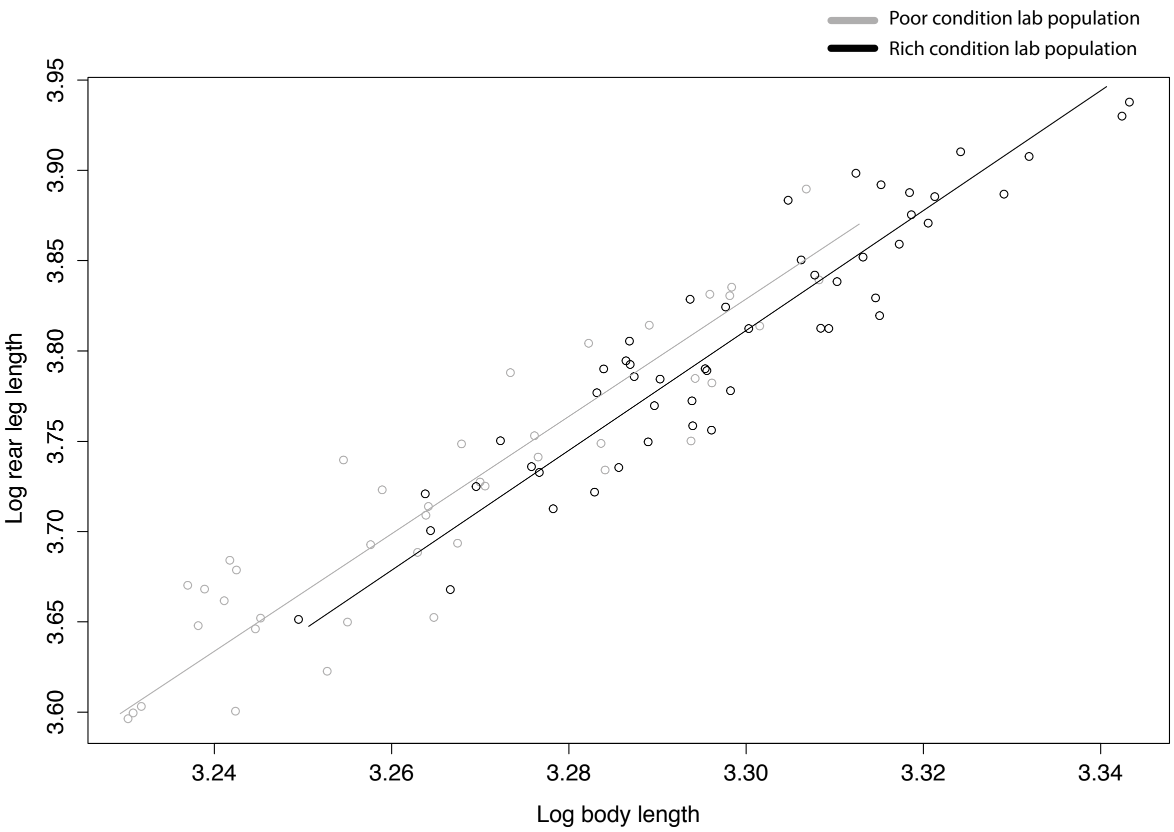

Supplementary figure 5: Nutrition effects on body-leg scaling relationships in the lab population. Static allometry on log-transformed data between rear leg and body lengths for unselected adult individuals fed on rich (black) and poor (grey) diets.

Supplementray tables' legends

Supplementary table 1: Morphometric data and associated statistical tests. Summary table of the adult measurements and statistical tests for all *Microvelia* species.

Supplementary table 2: Tests for normal distribution in all *M. longipes* conditions. Values of Shapiro tests and associated p-values for each *M. longipes* adult population reared in different conditions.

Supplementary table 3: Phylogenetic signals. Summary table of all tested characters and associated statistical tests of the phylogenetic signals.

Supplementary table 4: Summary table male competition in *M. longipes*. Table indicating the leg lengths, winning success and the group of all males used in the experiment (see methods for more details). Associated statistics are also reported.

Supplementary table 5: Fight frequency between *M. longipes* and *M. pulchella* males. Summary table of the number of fights in *M. longipes* and *M. pulchella* males in the different conditions for a period of one hour. Below are the associated statistical tests for differences in fight frequency.

Supplementary table 6: Counts and genotypes of the total number of eggs laid by females in each condition. A second excel sheet reports the tables of the summary statistics.

Supplementary table 7: Statistical tests associated with differences in leg length and scaling relationships between artificially selected and nutritionally manipulated *M. longipes* populations.

Supplementary table 8: Table containing the primer sequences, the full sequence, the motif and the length of each tested microsatellite.

Supplementary table 9: Table PCR protocols for microsatellite amplifications and single individual genotyping.

Supplementary table 10: Accession numbers of the 14 molecular marker sequences used in the phylogeny.

Supplementary videos' caption

Supplementary video 1: *M. longipes* male vibrations in slow motion.

Supplementary video 2: *M. longipes* mating system.

Supplementary video 3: *M. pulchella* mating system.

Supplementary video 4: *M.* sp. mating system.
